# Dynamic vs. Rigid: Transforming the Treatment Landscape for Multisegmental Lumbar Degeneration

**DOI:** 10.3390/jcm14155472

**Published:** 2025-08-04

**Authors:** Caner Gunerbuyuk, Mehmet Yigit Akgun, Nazenin Durmus, Ege Anil Ucar, Helin Ilkay Orak, Tunc Oktenoglu, Ozkan Ates, Turgut Akgul, Ali fahir Ozer

**Affiliations:** 1 Spine Center, Koc University Hospital, 34010 Istanbul, Turkey; drcaner@yahoo.com (C.G.); tuncoktenoglu@gmail.com (T.O.); atesozkan@hotmail.com (O.A.); alifahirozer@gmail.com (A.f.O.); 2 Department of Neurosurgery, Koc University Hospital, 34010 Istanbul, Turkey; dnazenin19@gmail.com (N.D.); eucar17@ku.edu.tr (E.A.U.); horak19@ku.edu.tr (H.I.O.); 3 Biomechanics and Endurance Laboratory, Koc University Research Center for Translational Medicine, 43606 Istanbul, Turkey; 4 Department of Orthopaedics and Traumatology, Istanbul University, 34730 Istanbul, Turkey; trgtakgul@gmail.com; 5 Bioengineering and Orthopaedic Surgery Colleges of Engineering and Medicine, University of Toledo, Toledo, OH 43606, USA

**Keywords:** multisegment, degenerative disc disease, dynamic, stabilization

## Abstract

**Background:** Multisegmental lumbar degenerative disease (ms-LDD) is a common condition in older adults, often requiring surgical intervention. While rigid stabilization remains the gold standard, it is associated with complications such as adjacent segment disease (ASD), higher blood loss, and longer recovery times. The Dynesys dynamic stabilization system offers an alternative by preserving motion while stabilizing the spine. However, data comparing Dynesys with fusion in multisegmental cases are limited. **Objective:** This study evaluates the clinical and radiographic outcomes of Dynesys dynamic stabilization versus rigid stabilization in the treatment of ms-LDD. **Methods:** A retrospective analysis was conducted on 53 patients (mean age: 62.25 ± 15.37 years) who underwent either Dynesys dynamic stabilization (n = 27) or PLIF (n = 26) for ms-LDD involving at least seven motion segments. Clinical outcomes were assessed using the Visual Analog Scale (VAS) and Oswestry Disability Index (ODI), while radiological parameters such as lumbar lordosis (LL), sagittal vertical axis (SVA), and spinopelvic parameters (pelvic incidence, pelvic tilt and, sacral slope) were analyzed. A two-stage surgical approach was employed in the Dynesys group to enhance osseointegration, particularly in elderly osteoporotic patients. **Results:** Both groups showed significant improvements in VAS and ODI scores postoperatively (*p* < 0.001), with no significant differences between them. However, the Dynesys group demonstrated superior sagittal alignment correction, with a significant increase in LL (*p* < 0.002) and a significant decrease in SVA (*p* < 0.0015), whereas changes in the rigid stabilization group were not statistically significant. Additionally, the Dynesys group had fewer complications, including a lower incidence of ASD (0 vs. 6 cases). The two-stage technique facilitated improved screw osseointegration and reduced surgical risks in osteoporotic patients. **Conclusions:** Dynesys dynamic stabilization is an effective alternative to rigid stabilization in ms-LDD, offering comparable pain relief and functional improvement while preserving motion and reducing ASD risk. The two-stage approach enhances long-term stability, making it particularly suitable for elderly or osteoporotic patients. Further long-term studies are needed to confirm these findings.

## 1. Introduction

Multisegmental lumbar degenerative pathologies are frequently seen in spinal surgery, predominantly affecting older adults [1]. Research by Boden et al. [2] has indicated that disc degeneration at an average of three levels can be observed in up to 90% of individuals over 60 years old. Lumbar fusion surgery has been the gold standard for these conditions for decades [3,4]. However, this procedure can lead to complications such as pseudoarthrosis, nonunion, instrumentation failure, infection, donor site pain, and adjacent segment disease (ASDis) [5]. Additionally, lumbar fusion often does not preserve lumbar motion, restricting the movement of stabilized segments and increasing the load on adjacent segments. This can raise the risk of adjacent segment degeneration (ASDeg), ASDis, and significant postoperative functional impairments [6]. The annual rate of surgical interventions for ASDis post-fusion is reported to be 3.9%, with a cumulative rate of 25–35% after 10 years [7].

In contrast to the fusion and instrumentation, the Dynesys Spinal Stabilization System stabilizes the treated segment while allowing some mobility, thereby maintaining more lumbar motion than fusion. Dynamic stabilization with the Dynesys system shows promise in improving patient outcomes and mitigating complications associated with ASDs, offering more physiological stabilization than traditional methods [8].

The Dynesys system is well-established, with numerous follow-up studies demonstrating positive outcomes such as short hospital stay, less blood loss, a quick return to daily life, etc., for various degenerative spinal conditions [9,10,11,12,13,14,15]. However, there is a scarcity of high-quality studies comparing Dynesys to fusion in terms of patient symptoms for multisegmental degenerative disease. Most existing studies focus on two-segment degenerative diseases, leaving a gap in the literature for cases involving more than two segments. We find this as a significant point for research because, as the number of fused segments increases, adjacent segments are more likely to experience greater axial loading and faster degeneration, leading to higher morbidity from ASDis.

In this retrospective study, we evaluated the clinical and radiographic outcomes of Dynesys dynamic stabilization compared to posterior lumbar interbody fusion (PLIF) in the treatment of multisegmental lumbar degenerative disease, including the use of a novel two-stage technique in the dynamic group aimed at enhancing osseointegration in elderly or osteoporotic patients.

## 2. Materials and Methods

### 2.1. Patient Selection Criteria

In this study, all procedures conducted adhered to the ethical guidelines set by both the institutional and national research committee, following the principles outlined in the 1964 Helsinki Declaration and its subsequent revisions or similar ethical standards. Prior informed consent was obtained from all participants who were part of the study.

A retrospective analysis was conducted on 53 patients treated with either dynamic or rigid stabilization for multisegmental degenerative diseases at the American and Koc University Hospitals between 2019 and 2022. Patient enrollment was consecutive during the study period. Inclusion criteria were as follows:

- Age ≥ 45 years;
- Radiologically confirmed multisegmental lumbar degenerative disease involving at least seven motion segments, demonstrated on MRI and standing whole-spine radiographs;
- Presence of chronic low-back pain lasting > 6 months with or without associated neurogenic claudication or radicular symptoms;
- Failure of at least 6 months of conservative management, including physical therapy, nonsteroidal anti-inflammatory drugs (NSAIDs), epidural steroid injections, and activity modification;
- Demonstrable dynamic instability on flexion–extension X-rays or instability evidenced by CT (e.g., facet joint fluid, vacuum phenomena, subchondral sclerosis);
- Functional impairment with Oswestry Disability Index (ODI) ≥ 30% preoperatively;
- A minimum postoperative follow-up of 24 months.

Patients with active infection, neoplastic lesions, prior extensive spinal instrumentation, or severe fixed deformities that were uncorrectable on dynamic imaging were excluded.

Dynamic stabilization using the Dynesys^®^ system was performed on 27 patients, while rigid stabilization was applied to 26 patients.

Instability was assessed subjectively by the presence of mechanical axial pain that worsened with activity and improved with rest. Objective radiological assessment included the following:

- Flexion–extension radiographs demonstrating sagittal translation > 3 mm or segmental angulation > 10° between vertebrae, consistent with established instability criteria;
- CT findings suggestive of segmental instability, such as vacuum phenomena within the intervertebral disc or facet joints, subchondral sclerosis, and hypertrophy of ligamentum flavum;
- MRI indicators, including hyperintensity or effusion within facet joints and Modic changes at endplates adjacent to the degenerated disc.

These radiological findings were correlated with the patient’s clinical presentation to confirm a diagnosis of functional spinal instability.

### 2.2. Surgical Technique

When deciding on dynamic stabilization, there should be no overt instability in the spine, and the deformity should be mobile or associated with slowly developing degenerative disease. The Silva–Lenke (SL) and Berjano–Lamartina (BL) classifications are used for adult spinal deformity patients. These classifications are typically applied for rigid stabilization but can be repurposed for dynamic stabilization [16]. According to a recent study, dynamic stabilization can be applied to patients classified as SL level 2–3 and BL type 1–3, and some cases of type 4 [16]. For the remaining patients, rigid stabilization is preferred.

All patients underwent surgery under general anesthesia in the prone position through a posterior-only approach. All surgeries were performed by the same team using the same technique in each group. Prophylactic cefazolin was given to all patients at the start of anesthesia and continued for the next 7–10 days. Surgical area was determined with fluoroscopy control.

For the dynamic stabilization group, surgery is performed in two stages. Firstly, Dynesys screws were inserted bilaterally at the levels under C-arm (fluoroscopy) guidance with the Wiltse method. In the first stage of the Dynesys procedure, pedicle screws were inserted, but the elastic cord–screw interface (‘thread’) was deliberately not tensioned or locked until the second stage to allow initial osseointegration under minimal mechanical load. Threads were inserted as a second surgery in 4–6 months after first operation. This 2-stage technique is preferred for elderly osteoporotic patients in practice. In 10 of the 27 Dynesys cases, we utilized O-ARM and navigation systems due to specific anatomical challenges—such as prior instrumentation, significant osteopenia, or deformity—where freehand technique risk was higher. In the remaining cases, standard fluoroscopy was deemed sufficient.

All PLIF procedures used a combination of local autograft harvested during decompression and demineralized bone matrix (DBM) (commercially sourced from Osteotech, Eatontown, NJ, USA).

On the other hand, for the rigid stabilization group, primarily transpedicular screws then titanium rods were placed, again with the guidance of C-arm, in the one stage surgery. The transverse processes, facet joints, and laminae of the adjacent stabilized levels were then decorticated, followed by posterolateral and interbody fusion with artificial bone grafts. Patients were mobilized on the first day after surgery and were discharged within 4 to 5 days. Postoperative CT was performed for all patients before discharge and during the follow-up period.

### 2.3. Clinical and Radiological Follow Up

Demographic details, including preoperative neurological status, pain scores, and radiological data, were collected along with intraoperative information such as blood loss, surgery duration, and complications. Postoperative data, including neurological status, hospital stay duration, and pain scores, were also examined. All patients underwent two-way whole spine X-rays, MRI, and CT scans to guide the surgical plan based on individual clinical evaluations. Patients were closely monitored both clinically and radiologically at 6 and 12 months postoperatively after a thorough preoperative assessment. Measurements included segmental lordosis, lumbar lordosis, thoracic kyphosis angle, sagittal vertical axis (SVA), and pelvic parameters (pelvic incidence, pelvic tilt, and sacral slope). Patients were then scheduled for annual routine follow-ups. Clinical assessments involved neurological evaluations, and the Visual Analog Scale (VAS) and Oswestry Disability Index (ODI) were used for subjective patient evaluations preoperatively and at 6, 12, and 24 months postoperatively.

### 2.4. Statistical Analysis

Statistical analyses were performed using SPSS software (version 22, SPSS Inc., Chicago, IL, USA) and GraphPad Prism (version 9). Continuous variables were expressed as mean ± standard deviation. The paired-samples *t*-test was used to compare preoperative and postoperative values within each group, as the data were normally distributed and measurements were repeated on the same individuals. The independent-samples *t*-test was used for intergroup comparisons. For comparisons involving more than two time points, repeated-measures ANOVA was applied to evaluate trends over time. Statistical significance was defined as *p* < 0.05. Levels of significance were reported as follows: *p* < 0.05 (*), *p* < 0.01 (**), *p* < 0.001 (***), and *p* < 0.0001 (****).

## 3. Results

A total of 53 patients were included in the study, consisting of 22 women (41.5%) and 31 men (58.5%) with a mean age of 62.25 ± 15.37 years (range: 45–87 years). Among these, the Dynesys system was used in 27 patients (50.94%), and rigid stabilization was used in 26 patients (49.05%). The mean follow-up period was 38.12 months (range: 25–53 months). Thirteen patients had involvement in seven segments, thirteen patients had involvement in eight segments, twelve patients had involvement in nine segments, and fifteen patients had involvement in ten segments (Table 1).

Preoperative and postoperative radiological parameters were evaluated for each group. The Dynesys group demonstrated superior sagittal alignment correction, with a mean increase in lumbar lordosis (LL) of 12.44° (from 21.19° to 33.63°, *p* < 0.002) and a mean decrease in sagittal vertical axis (SVA) of 19.55 mm (from 36.44 mm to 16.89 mm, *p* < 0.0015), indicating a markedly more pronounced correction In contrast, the rigid group showed a non-significant decrease in LL of 6.2° (from 31.50° to 25.30°) and a slight increase in SVA of 2.8 mm (from 20.50 mm to 23.30 mm). However, there was no significant difference in postoperative lumbar lordosis between the two groups.

The reduction in SVA in the dynamic stabilization group, from a mean of 36.44 mm preoperatively to 16.89 mm at the final follow-up (24 months postoperatively), was statistically significant (*p* < 0.0015). In contrast, the rigid stabilization group demonstrated a slight increase in SVA from 20.50 mm to 23.30 mm over the same period, which was not statistically significant (*p* = 0.9870).

A possible explanation for the greater SVA correction in the dynamic group may relate to the younger average surgical levels and the two-stage approach allowing a gradual correction of sagittal alignment over time, especially in osteoporotic patients. The preservation of segmental motion may have facilitated adaptive postural compensation, enhancing sagittal balance postoperatively. Conversely, in the rigid stabilization group, the fusion construct may have restricted compensatory mechanisms, particularly in long-segment cases where rigidity can limit pelvic retroversion and spinal flexibility.

Segmental lordosis and thoracic kyphosis values showed no significant changes in either the dynamic stabilization group (*p* = 0.7824 and *p* = 0.3077, respectively) or the rigid stabilization group (*p* = 0.4150 and *p* = 0.9664, respectively).

Although pelvic parameters (PI, PT, SS) were recorded, no statistically significant differences were observed between groups postoperatively.

Among the 53 patients, a total of 8 patients (15%) had a history of prior lumbar spine surgery and underwent revision procedures as part of this study. Of these, 4 patients were in the dynamic stabilization group and 4 patients were in the rigid stabilization group. Prior surgeries included single-level decompression or fusion at L3–L5 in most cases. These patients were included due to either the progression of degenerative disease at adjacent or remote segments or the failure of previous intervention. Revision cases were equally distributed and did not significantly impact the comparative outcomes between groups.

Clinical outcomes were evaluated at 3, 6, 12, and 24 months postoperatively. Both the Visual Analog Scale (VAS) and the Oswestry Disability Index (ODI) scores demonstrated significant improvements postoperatively in both groups (*p* < 0.001); however, no statistically significant difference was found between the groups (Table 2 and Table 3).

Perioperative parameters, including total surgery time, intraoperative blood loss, and duration of hospital stay, were evaluated for both groups and are summarized in Table 4. For the Dynesys group, values represent the cumulative total of both surgical stages. Despite the staged nature of the procedure, patients in the Dynesys group had significantly shorter operative time, lower blood loss, and shorter overall hospitalization compared to the rigid stabilization group. However, we did not perform a formal cost analysis in this study.

The complications are follows:

- Dynesys group: Thread breakage occurred in 2/27 patients (7.4%);
- Rigid group: Instrument failure in 2/26 (7.7%), ASD in 6/26 (23.1%), pseudoarthrosis in 2/26 (7.7%), infection in 1/26 (3.8%), and 1 case (3.8%) of postoperative neurological deficit.

All cases in both the dynamic and rigid stabilization groups required revision surgery, except for one ASD case in the rigid stabilization group, which did not undergo revision. Additionally, one patient in the rigid stabilization group experienced postoperative neurological deficit and, in both groups, one patient experienced an infection, though neither required further surgical intervention.

**Figure 1:**
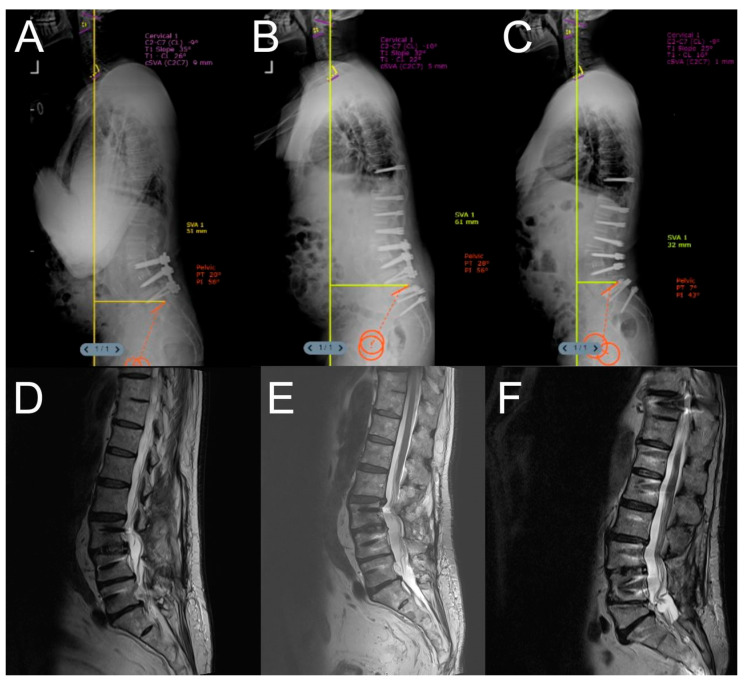
A patient who had previously undergone L3–4 interbody fusion and L3–5 stabilization for spondylolisthesis presented with difficulty looking forward and increased kyphosis. (**A**) Preoperative lateral X-ray image. (**B**) Lateral X-ray image after the first-stage surgery. (**C**) Lateral X-ray image after the second-stage surgery. (**D**) Sagittal MRI image after the initial L3–5 stabilization. (**E**) Preoperative sagittal MRI image showing proximal junctional kyphosis and Pfirrmann grade 4 intervertebral disc degeneration. (**F**) Postoperative sagittal MRI image after the second-stage surgery. Due to the patient’s osteoporosis (T-score = −2.5 preoperatively, T-score: −1.5 before the two-stage surgery), a two-stage surgical approach was adopted. Significant improvement was observed in both clinical findings and spinopelvic parameters in the postoperative period.

**Figure 2:**
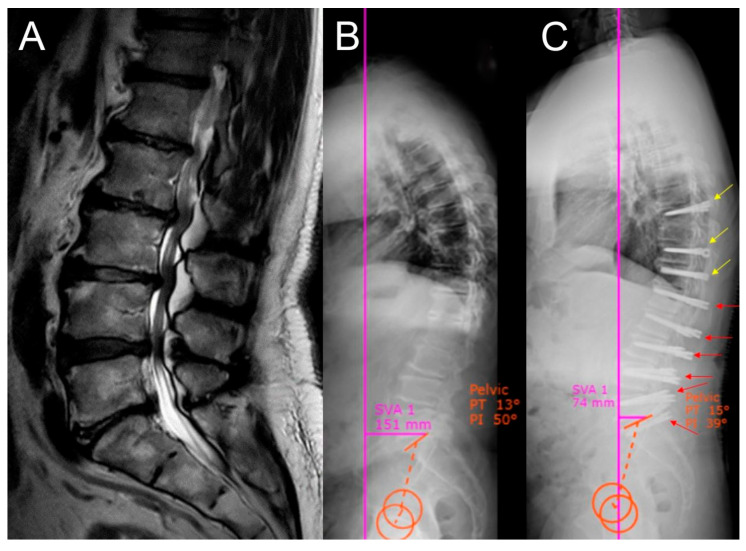
Radiological images of a patient presenting with walking difficulty and low back pain. Decompression and dynamic stabilization using Dynesys were performed. (**A**) Preoperative sagittal MRI image showing Pfirrmann grade 4 intervertebral disc degeneration. (**B**) Preoperative lateral X-ray image showing sagittal imbalance and pelvic parameters. (**C**) Postoperative lateral X-ray image showing improvement in sagittal balance.

**Figure 3:**
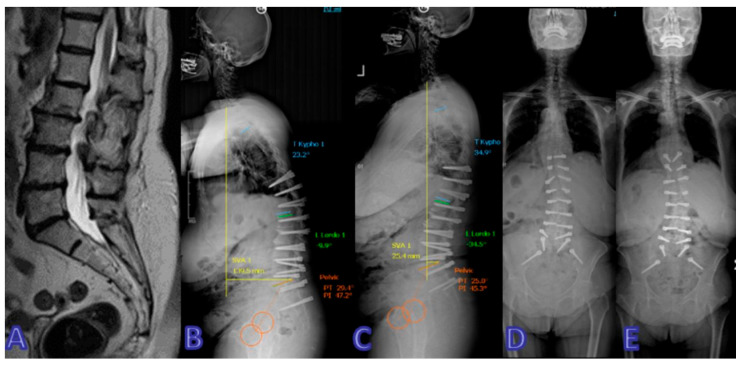
A 69-year-old woman underwent surgery for L4–5 stenosis 10 years ago and presented with progressive forward bending and low-back pain. (**A**) Preoperative MRI image. (**B**) Lateral X-ray image after the first stage (prior to rod placement). (**C**) Lateral X-ray image after the first stage (following rod placement). (**D**) Anteroposterior X-ray image after the first stage (prior to rod placement). (**E**) Anteroposterior X-ray image after the first stage (following rod placement). Significant improvement was noted in both clinical findings and spinopelvic parameters postoperatively.

**Figure 4:**
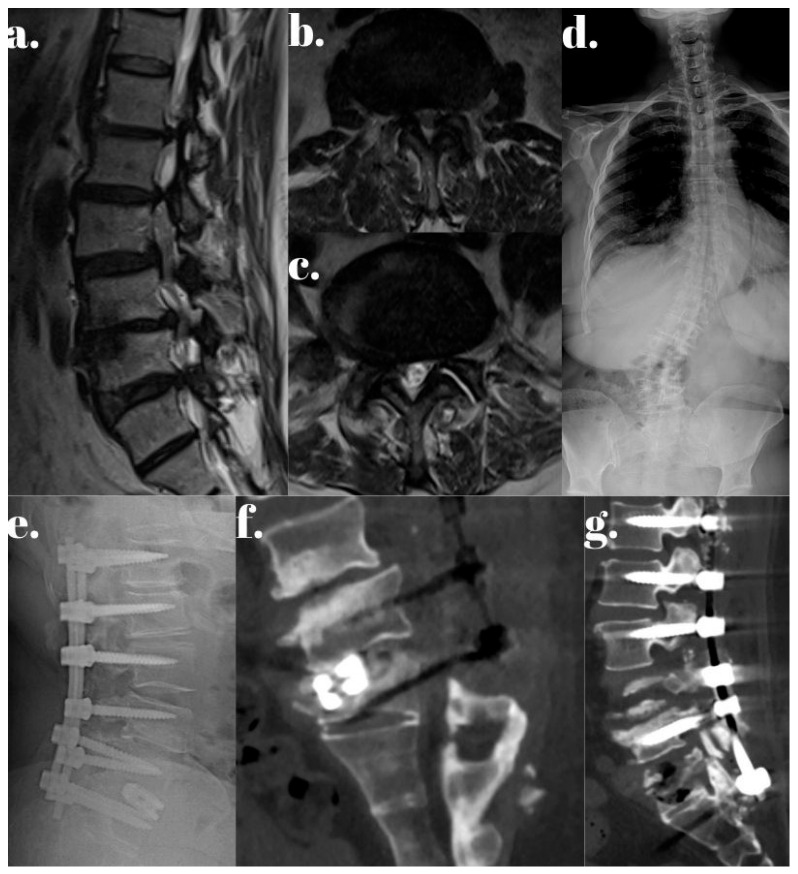
A patient who underwent L1–S1 rigid stabilization with TLIF for severe low back pain experienced worsening low-back pain at six months postoperatively. CT imaging revealed TLIF subsidence and S1 screw malposition. (**a**) Preoperative sagittal MRI image. (**b**,**c**) Preoperative axial MRI images. (**d**) Preoperative anteroposterior X-ray image. (**e**) Lateral X-ray image after the initial L1-S1 rigid stabilization with TLIF. (**f**) Sagittal CT image showing TLIF subsidence and S1 screw malposition. (**g**) Sagittal CT image following revision surgery (2nd year postoperatively).

**Figure 5:**
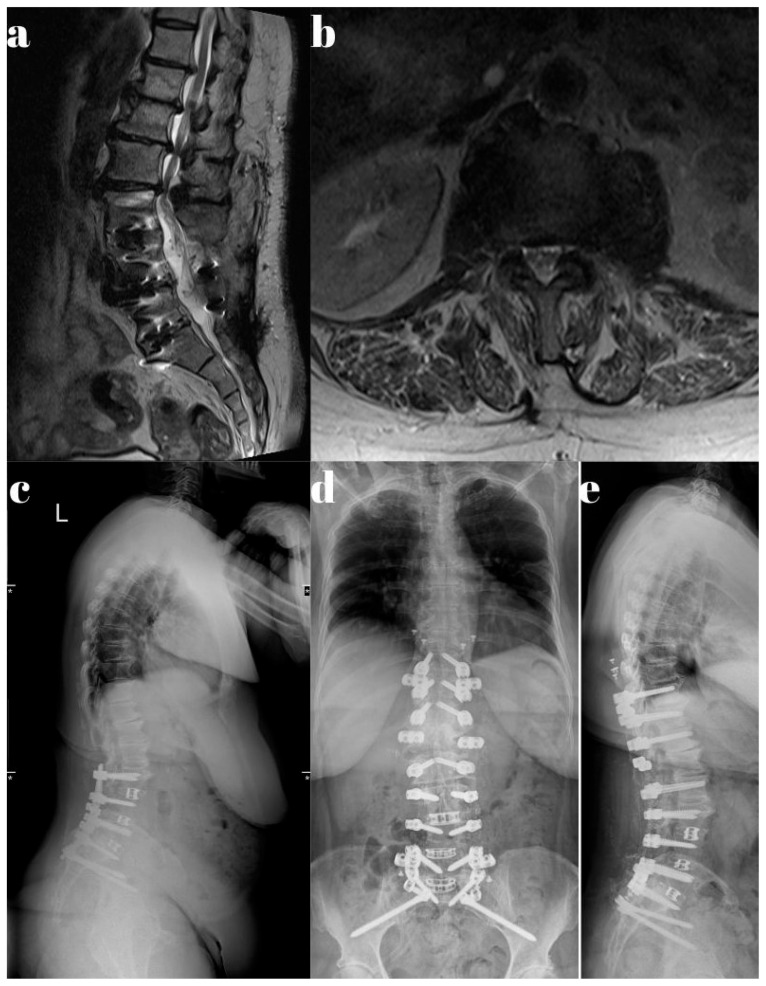
A patient who previously underwent L3-iliac rigid stabilization developed adjacent segment disease and presented with severe left leg and back pain. MRI revealed multilevel stenosis from T11–L3. (**a**) Sagittal T2-weighted MRI showing multilevel spinal stenosis. (**b**) Axial T2-weighted MRI demonstrating foraminal stenosis. (**c**) Preoperative full-spine lateral X-ray showing L3-iliac rigid stabilization with three-level TLIF instrumentation. (**d**) Postoperative full-spine coronal X-ray displaying dynamic stabilization. (**e**) Postoperative full-spine lateral X-ray demonstrating dynamic stabilization.

Representative cases from both the Dynesys and rigid stabilization groups are illustrated in Figure 1, Figure 2, Figure 3, Figure 4 and Figure 5.

## 4. Discussion

The incidence of multisegmental lumbar spinal stenosis (ms-LSS) is rising, but its optimal treatment remains debated. While multi-segmental decompression alone may lead to spinal instability, lumbar fusion is the most common procedure to maintain stability. However, lumbar fusion carries risks of greater blood loss and higher perioperative complications [17]. Treating ms-LSS with fusion surgery poses challenges, particularly for elderly patients due to their lower tolerance, the need for bone grafting materials, high costs, increased surgical trauma, and higher blood loss. Additionally, as the number of fused segments increases, adjacent segments often experience higher axial loading and accelerated degeneration, raising the incidence of adjacent segment disease (ASD) [18].

Rigid stabilization has been accepted as the gold standard surgical treatment for many years for multiple reasons. For a long time, there was no alternative to this procedure, but it works very well for many patients despite its associated complications. Firstly, if a patient has SL level 4–6 or certain cases of BL type 4, dynamic systems cannot be used. Additionally, if the deformity is fixed and does not correct spontaneously with changes in the patient’s position, rigid stabilization becomes inevitable. To evaluate whether the deformity is mobile or not, lying and standing radiographs can be used. A fixed deformity may result from delayed intervention, as the spine’s compensatory mechanisms can become exhausted. Furthermore, if the deformity develops rapidly, rigid stabilization should be preferred.

The Dynesys system offers advantages in managing ms-LSS, particularly in elderly patients, as it avoids the need for bone grafting, potentially minimizes surgical trauma, reduces blood loss, and allows for faster recovery [19]. Dynamic stabilization also reduces operation time, as it does not require fusion [8,20]. Unlike posterior lumbar interbody fusion (PLIF), Dynesys can support and balance the load between vertebrae while preserving the motor function of the stabilized segments, thereby mitigating the stress and movement impacts on adjacent segments, and potentially preventing or delaying ASD. Theoretically, Dynesys dynamic stabilization offers more advantages over PLIF. This retrospective case–control study compared the clinical outcomes and radiographic features of Dynesys stabilization versus PLIF for treating multisegmental lumbar degenerative disease [1,3].

While the Dynesys system has been in clinical use for more than two decades and is well-studied for short-segment degenerative disease, its application in multisegmental (≥7 levels) dynamic stabilization remains relatively novel, with limited long-term outcome data. Most published studies focus on 1- or 2-level constructs, and evidence supporting Dynesys in extended segmental disease is still emerging. While dynamic systems come with many advantages, which will be discussed in later paragraphs, the most feared complication in long-term follow-up is instrument failure, especially when applied to long segments. Dynesys and other dynamic systems work very well in short-segment stabilization, but long-term evidence is still lacking. Therefore, patient selection is crucial to avoid this significant complication.

Dynamic stabilization with the Dynesys system has been found to be less invasive than instrumented posterior fusion, with benefits including shorter hospital stays and reduced blood loss [17,19]. Multiple clinical studies have shown that patients with degenerative lumbar disease treated with the Dynesys system experience significant improvements in Oswestry Disability Index (ODI) and Visual Analog Scale (VAS) pain scores, as well as faster recovery times, compared to those who undergo fusion [11,21,22,23,24,25,26]. In this study, both dynamic and rigid stabilization groups exhibited significant improvements in ODI and VAS scores at the final follow-up compared to their baseline values (*p* < 0.05). However, there was no significant difference between the two groups’ scores at the final follow-up (*p* > 0.05). When we analyzed the surgery time, blood loss, and hospital stay, dynamic stabilization was superior to the fusion systems with shorter surgery time, less blood loss, and shorter hospital stay. These findings are consistent with those reported in the existing literature.

In our series of dynamic stabilization cases, a two-stage surgical approach is preferred. This technique is chosen for several reasons. Firstly, the mean age of the patient population in the dynamic stabilization group is 64.26 years. This age group is often characterized by osteoporosis, high body mass index (BMI), and muscle atrophy associated with fatty degeneration. These conditions can facilitate the insertion of screws; however, they compromise the osseointegration of screws, increasing the mechanical load on the instrumentation and rendering screw loosening inevitable.

To mitigate this complication, we initially insert screws percutaneously into the vertebrae without placing spacers. This ensures that the screws are not subjected to body weight and allows them to fuse with the bone over time (typically within 4–6 months). Before proceeding with the second stage, a computed tomography (CT) scan is performed to confirm osseointegration. Once confirmed, spacers are placed on the screws and fixed without difficulty.

It is important to note that this technique is only suitable for patients who do not experience unbearable pain, as a 4–6-month interval is required between the two stages. This patient population generally presents with minor symptoms that allow them to manage their daily lives during this period. Our approach is inspired by implant technology widely employed in dentistry [27,28,29].

Prior to surgery, a patient’s T-score should be determined using dual-energy X-ray absorptiometry (DEXA). If the T-score is less than −1.5, the two-stage surgical approach is deemed applicable. Additionally, dividing a complex stabilization procedure into two simpler operations makes the surgery more tolerable for elderly patients, who often have multiple comorbidities. This approach also reduces the likelihood of future revision surgeries [30].

The restoration of sagittal spinal balance is another critical factor, as it reduces the incidence of adjacent segment disease and screw loosening [31]. While the two-stage surgery approach minimizes these complications, ensuring proper sagittal alignment is equally crucial. This finding aligns with our study, which demonstrates significant improvements in lumbar lordosis (LL) and sagittal vertical axis (SVA) within the dynamic stabilization group.

Although our study did not find statistically significant differences in Visual Analog Scale (VAS) and Oswestry Disability Index (ODI) scores between the two groups, the existing literature suggests that proper sagittal spinal balance is strongly correlated with reduced low-back pain both preoperatively and postoperatively. When patient alignment is correctly addressed, VAS and ODI scores tend to show statistically significant improvements [31]. This discrepancy may be attributable to the number of spinal levels included in the stabilization procedure.

Thus, we conclude that preoperative sagittal balance significantly influences surgical outcomes. If surgeons can achieve optimal spinal alignment during surgery, the results are markedly improved. In the dynamic stabilization group, spinopelvic parameters were corrected to a greater extent compared to the rigid stabilization group.

While our study focused on comparing Dynesys with PLIF, we acknowledge that minimally invasive techniques such as MIS-TLIF, LLIF, and percutaneous screw fixation are increasingly favored for spinal stabilization. These approaches are associated with reduced muscle trauma, blood loss, and recovery time. However, for long-segment degenerative disease (≥7 segments), MIS techniques are technically challenging and less frequently performed, especially in osteoporotic or deformed spines. Furthermore, Dynesys dynamic stabilization inherently uses a motion-preserving concept, which may offer biomechanical advantages over fusion, regardless of whether the fusion is performed via open or MIS techniques. We encourage future prospective comparisons between Dynesys and MIS fusion strategies in shorter-segment disease and selected multisegmental cases to further refine patient selection and technique optimization.

One important consideration is that dynamic stabilization in this study was performed using a two-stage surgical technique, which inherently involves longer overall treatment duration, potential for increased perioperative anxiety, and repeat anesthesia exposure. While this approach was adopted to enhance osseointegration and improve outcomes in elderly or osteoporotic patients, it may not be suitable for all patient populations. The cumulative burden of two separate surgeries should be weighed carefully in clinical decision-making. Additionally, because the indication for dynamic stabilization is more selective, particularly for patients with mobile deformities and no severe instability, this study does not represent a randomized controlled comparison. Selection bias may exist despite attempts to match baseline characteristics.

## 5. Limitations

This study has several limitations. First, its retrospective design may introduce selection bias, as patients were not randomly assigned to treatment groups. Second, the sample size is relatively small, which may limit the statistical power to detect differences between groups. In the literature, cases of long-segment dynamic stabilization are also limited, so this paper is considered a preliminary investigation. Third, the follow-up period, while sufficient for short- to mid-term outcomes, may not fully capture long-term complications such as screw loosening, implant failure, or adjacent segment disease progression. Additionally, our study did not include a control group undergoing conservative treatment, which could provide a better comparison for evaluating surgical outcomes. Finally, while radiological and clinical parameters were assessed, patient-reported outcomes beyond VAS and ODI, such as quality of life measures, were not included, which may limit the comprehensive evaluation of functional recovery. Future prospective studies with larger cohorts and longer follow-up periods are needed to validate these findings.

## 6. Conclusions

Dynamic stabilization with the Dynesys system demonstrates clear advantages in multisegmental lumbar degenerative disease by preserving spinal mobility, reducing complications like adjacent segment disease, and significantly improving sagittal alignment. The two-stage surgical approach enhances osseointegration and long-term stability, particularly in elderly or osteoporotic patients, while minimizing surgical risks. These benefits make Dynesys a promising alternative to traditional fusion techniques, offering effective and less invasive management for complex degenerative conditions.

## Figures and Tables

**Table 1:** Patients’ baseline data.

	Dynamic Stabilization Group (50.94%)	Rigid Stabilization Group (49.05%)	*p*-Value
Gender (male/female)	19/8	12/14	
Age (years)	64.26 ± 15.48	60.24 ± 15.52	0.367
Follow up (months)	30.25	43.41	<0.05
Surgical levels			
7	7	6	
8	6	7	
9	6		6
10	8		7

**Table 2:** VAS (Visual Analog Scale) scores pre- and postoperatively for Dynesys and rigid stabilization groups.

Time Point	Dynesys Group (Mean ± SD)	Rigid Stabilization Group (Mean ± SD)	*p*-Value
Preoperative	7.4 ± 1.2	7.5 ± 1.3	0.772
3 Months Postop	3.1 ± 2.0	3.0 ± 1.9	0.853
6 Months Postop	2.6 ± 1.8	2.7 ± 1.7	0.836
12 Months Postop	2.2 ± 1.5	2.4 ± 1.6	0.641
24 Months Postop	1.9 ± 1.4	2.1 ± 1.5	0.618

**Table 3:** ODI (Oswestry Disability Index) scores pre- and postoperatively for Dynesys and rigid stabilization groups.

Time Point	Dynesys Group (Mean ± SD)	Rigid Stabilization Group (Mean ± SD)	*p*-Value
Preoperative	45 ± 10	46 ± 11	0.731
3 Months Postop	25 ± 15	26 ± 14	0.803
6 Months Postop	22 ± 14	23 ± 13	0.788
12 Months Postop	18 ± 12	19 ± 12	0.763
24 Months Postop	16 ± 11	17 ± 12	0.753

**Table 4:** Comparison of perioperative parameters between Dynesys and rigid stabilization groups (cumulative values for Dynesys two-stage procedures).

Parameter	Dynesys Group (n = 27)	Rigid Stabilization Group (n = 26)	*p*-Value
Total Surgery Time (min)	180 ± 35 (combined stages)	230 ± 40	0.002
Blood Loss (mL)	350 ± 80 (combined)	520 ± 110	0.001
Hospital Stay (days)	5.8 ± 1.2 (cumulative)	7.3 ± 1.5	0.003

## Data Availability

The datasets generated during and/or analyzed during the current study are available from the corresponding author on reasonable request.
